# Presence and absence of intrinsic magnetism in graphitic carbon nitrides designed through C–N–H building blocks

**DOI:** 10.1038/s41598-022-05590-4

**Published:** 2022-02-11

**Authors:** Teerachote Pakornchote, Annop Ektarawong, Akkarach Sukserm, Udomsilp Pinsook, Thiti Bovornratanaraks

**Affiliations:** Extreme Conditions Physics Research Laboratory and Center of Excellence in Physics of Energy Materials(CE:PEM), Department of Physics, Faculty of Science, Chulalongkorn University, Bangkok, 10330 Thailand

**Keywords:** Magnetic materials, Spintronics, Structural properties, Density functional theory, Structure prediction

## Abstract

We use the first principle calculation to investigate the intrinsic magnetism of graphitic carbon nitrides (GCNs). By preserving three-fold symmetry, the GCN building blocks have been built out of different combinations between 6 components which are C atom, N atom, s-triazine, heptazine, heptazine with C atom at the center, and benzimidazole-like component. That results in 20 phases where 11 phases have been previously reported, and 9 phases are newly derived. The partial density of states and charge density have been analyzed through 20 phases to understand the origin of the presence and absence of intrinsic magnetism in GCNs. The intrinsic magnetism will be present not only because the GCNs comprising of radical components but also the $\pi$-conjugated states are not the valence maximum to break the delocalization of unpaired electrons. The building blocks are also employed to study alloys between g-$\hbox {C}_3\hbox {N}_4$ and g-$\hbox {C}_4\hbox {N}_3$. The magnetization of the alloys has been found to be linearly dependent on a number of C atoms in the unit cell and some magnetic alloys are energetically favorable. Moreover, the intrinsic magnetism in GCNs can be promoted or demoted by passivating with a H atom depending on the passivated positions.

## Introduction

Various types of 2-dimensional (2D) materials have been explored their magnetism that suite for spintronics and magnetic devices. For metal-free materials, 2D organic frameworks such as triangulenes and graphitic carbon nitrides (GCNs) are candidates that possess intrinsic magnetism with high Curie temperature and high flexibility^1–4^. Organic radicals can be linked by a triazine which is a carbon-nitride ring stabilizing paramagnetic 2D organic frameworks^5^. The triangulene which is a fragment of graphene can be scaled its magnetization by increasing a size of the fragment^6,7^. It can be crystallized by connecting its edges with other chemical groups^8^. For graphene, each C atom spends 3 electrons to form $\sigma$ bonds and leaves one electron forming conjugated $\pi$ bond suppressing graphene’s magnetism while the magnetism in triangulenes exists due to lack of $\pi$ conjugation. Accordingly, the magnetism of 2D organic frameworks can be suppressed by a delocalization of electrons in $\pi$ orbitals affected by compressive strain^9^.

The intrinsic magnetism in graphene can be risen by a defect producing a half-filled band of $\pi$ orbitals^10^. The defects can be either voids or impurities splitting the spin-up and spin-down $p_z$ states at the Fermi level ($\hbox {E}_F$) and inducing a magnetic moment^11–14^. One method used to create void defects in graphene is by doping N atoms where the voids are surrounded by N atoms, then N-doped graphene becomes magnetic^15,16^. Although, high doping, void, and passivation concentrations lead to phase instability limiting amounts of spin density in graphene^17–19^.

In this work, we focus on GCNs whose several magnetic and non-magnetic phases have been predicted. For instance, g-$\hbox {C}_4\hbox {N}_3$ is a magnetic phase with one Bohr magneton ($\mu _B$) per unit cell^20,21^, and $\hbox {C}_{{14}}\hbox {N}_{{12}}$ bears 2$\mu _B$ per unit cell of magnetization^4^. $\hbox {C}_{{9}}\hbox {N}_{{7}}$ and $\hbox {C}_{{10}}\hbox {N}_{{9}}$ are magnetic phases while $\hbox {C}_{{3}}\hbox {N}_{{2}}$, $\hbox {C}_{{4}}$N, and $\hbox {C}_{{9}}\hbox {N}_{{4}}$ are non-magnetic^4,15^. Their magnetism arises from an unpaired electron contributing a magnetic moment to a system. The structure that has two unpaired electrons can be non-magnetic or magnetic depending on they are in singlet or triplet states, respectively^22,23^. Anyhow, magnetic GCNs have not yet achieved in experiments. The GCN that is typically synthesized are g-$\hbox {C}_3\hbox {N}_4$, but it is non-magnetic. It is in a form of s-triazine or heptazine networks and a candidate for photocatalysis and carbon dioxide adsorbing materials^24–31^. Some extra-treatments have been used to activate the intrinsic magnetism of this phase^32,33^. For example, the magnetism in GCNs can be enhanced by defects, hydrogenation, and fluorination^34–40^. GCNs are particularly porous, so a metal atom can sit in a hole and induces magnetism of the structure^41,42^. The metal atom can also be a linkage connecting atoms and build blocks, but the Curie temperature is not as high as a light-atomic linkage^43,44^.

Herein, we present an aspect that structures of GCNs can be constructed from building blocks of C–N assisting us to understand the presence and absence of intrinsic magnetism of GCNs. The construction is focused on the building block whose two components are connecting together with three-fold symmetry. The first principle method based on spin-polarized density functional theory (DFT) has been employed to study thermodynamics stability, electronic structure and magnetism of GCNs. The absence and presence of intrinsic magnetism of GCNs have been analyzed through their electronic structures. We propose two mechanisms playing a crucial role in GCNs magnetism; firstly, one out of two components in the building block must have a free radical, and secondly, $\pi$ conjugation does not present near the Fermi energy if both components have free radicals. We also present a series of study on hydrogenated and alloy GCNs; the GCNs prefer to be passivated by a H atom at some locations able to activate or deactivate the intrinsic magnetism, and the magnetization of GCNs can be modified by mixing different building blocks.

## Results and discussion

### Building blocks

**Figure 1:**
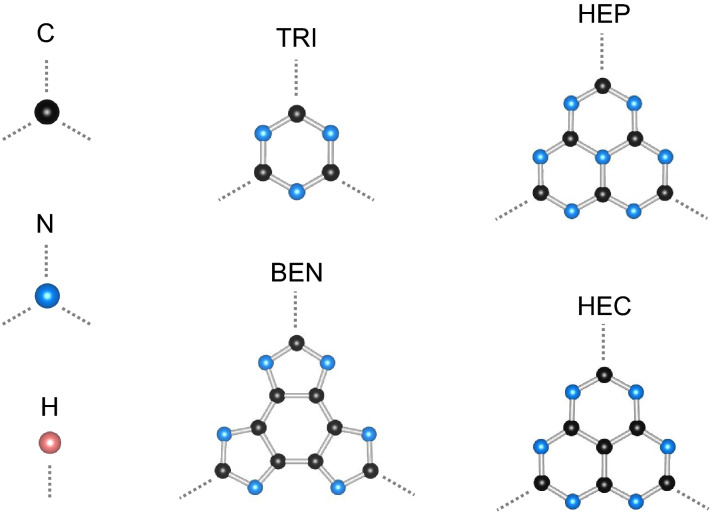
Components of building blocks with three-fold connecting parts except for H atom that has one connecting part shown as grey dotted lines.

The building blocks are C, N, H, s-triazine (TRI), heptazine (HEP and HEC) and tetracyclic benzimidazole (BEN) components where HEC is a heptazine with C atom at the center instead of N atom (see Fig. 1). Each component will be joint with other components in three directions preserving three-fold symmetry except for H atom that will be only dangling with C and N atoms. One can think of an alloy which is a mixture of several components; however, this work is limited to study connections between two types of components where the first component is a *core*, and the second component is a *connector*. Therefore, in this study, GCNs are constructed from cores which are TRI, HEP, HEC and BEN, and connectors which are C, N, TRI, and HEP while a H atom is neither a core nor a connector but a passivating atom. GCNs are typically labeled by a tuple of numbers of C and N atoms, ($n_\text {C}$, $n_\text {N}$), in a unit cell which is sometimes ambiguous, so they are, instead, designated as *core-connector*, e.g. TRI-N whose common name is g-$\hbox {C}_3\hbox {N}_4$ is a structure where TRI is a core and N is a connector.

By our constructions, there are 20 phases reported here; 11 phases, to the best of our knowledge, have been reported before^4,20,21,24–29,29,45–52^, and 9 phases which are HEP–TRI, HEP–HEP, HEC-C, HEC–HEP, and BEN-X (X = C, TRI, HEP, HEC, and BEN) are newly derived by this study (see Fig. S1 in Supplementary Information). Table 1 presents the formation energy ($E_{form}$) of all phases which are entirely positive in the order of hundreds meV by comparing with the energy of graphene and $\hbox {N}_2$ molecule. Noting that GCNs can be synthesized from chemical compounds, e.g., melamine, melam, and melon, so their $E_{form}$ would change according to precursors.

Figure S2 in Supplementary Information shows that the $E_{form}$ seems to decrease with respect to N concentration. However, it is not a good presentation because different types of GCNs are being compared. Bu et al.^19^ shows that the formation energy of N-doped graphene and GCNs with pyridinic N increase similarly with amount of N concentration up to 0.25%. The former starts increasing higher if N concentration is more than 0.3%. The structures with pyrrolic N also have formation energy higher than the structures with pyridinic N. In this work, TRI, HEP, and HEC are components with pyridinic N, while BEN is a component with pyrrolic N. The formation energy of structures with BEN component is thus higher than that of structures without BEN component. The formation energy of structures with HEP component are lower than that of structures with HEC component even HEP is a N-doped HEC. The structures with N connector are more energetically favorable than those with C connector.

TRI–TRI has the highest $E_{form}$ among other phases. The C–N–C angle of TRI component is ideally 120$^{\circ }$. It is 108.4$^{\circ }$ in TRI–TRI while it is about 114$^{\circ }$ to 120.5$^{\circ }$ in other structures whose the TRI component is constituent. This angle in TRI–TRI that is much sharper than other phases may cause high strain on the structure throwing the $E_{form}$ of TRI–TRI to 1.368 eV.

**Table 1:**
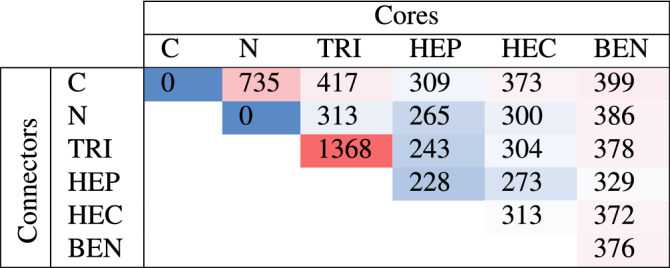
The $E_{form}$ of each GCN is reported in meV per atom unit where each column and row are core and connector components, respectively. The blue–white–red colors are shaded according to the values from lowest to highest. N–N, in contrast, denotes a $\hbox {N}_2$ molecule.

### Magnetic and electronic properties

The intrinsic magnetism in GCNs is present or absent due to combinations between cores and connectors. Table 2 presents the magnetizations of GCNs where each phase is constructed from the core and the connector labeled in each column and row, respectively. Since the core and the connector are commutable, the values below diagonal should be the same as the values above diagonal. Most phases, whose intrinsic magnetism are present, have either HEC or BEN as components except for HEC-C, BEN-C and BEN–BEN that are non-magnetic. Two more magnetic phases are TRI-C and HEP-C. The magnetism is absent in C–C, C–N, TRI-N, HEP-N and HEP–HEP where C–C and C–N are graphene and 2D honeycomb carbon nitride, respectively. Noting that N–N is a $\hbox {N}_2$ molecule, so it is disregarded.

We count a number of valence electrons, which do not form $\sigma$ bonds, per unit cell ($n_e$) of each phase in order to understand a key factor governing presence or absence of the intrinsic magnetism. In a honeycomb structure, a C atom covalently bonds with its three nearest atoms leaving one lone electron, while a N atom leaves two electrons which is a lone pair. In TRI, HEP, HEC, and BEN, the pyridinic and pyrrolic N atoms bonding with only two nearest atoms have $n_e=3$; therefore, TRI and HEP have even $n_e$ considered to be non-magnetic components, and HEC and BEN have odd $n_e$ considered to be magnetic components. We expect that if the $n_e$ is even, all electrons left over from $\sigma$ bonding will pair together suppressing the intrinsic magnetism of the structure. In contrast, if the $n_e$ is odd, a single electron will be a free radical granting a magnetic moment. The parity of the $n_e$ of each phase is presented in Table 2 and labeled in blue if the $n_e$ is odd and yellow if the $n_e$ is even. As labeled, the GCNs are magnetic or non-magnetic if the $n_e$ are odd or even, respectively, except for C–N, HEC–HEC and BEN–HEC. The magnetic behavior of the excepted phases, in contrast, can be explained through the projected DOS (PDOS) and the localization of the charge density. The intrinsic magnetism of GCNs is then discussed separately for each core component in subsections below. The PDOS will be shown by types of atoms where $\hbox {C}_{{A}}$ and $\hbox {N}_{{A}}$ denote C and N atoms of the A component, respectively.

**Table 2:**
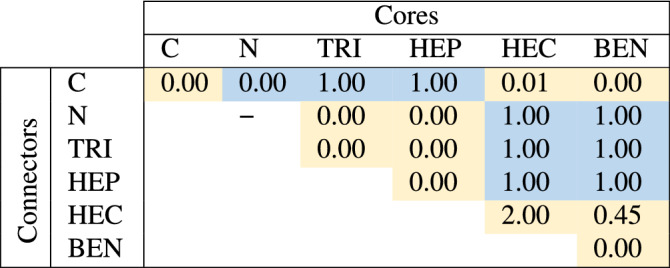
The magnetization of each GCN is reported in $\mu _B$ unit where each column and row are core and connector components, respectively. The parity of the $n_e$ of each phase is labeled by blue and yellow denoting odd and even, respectively.

**Table 3:**
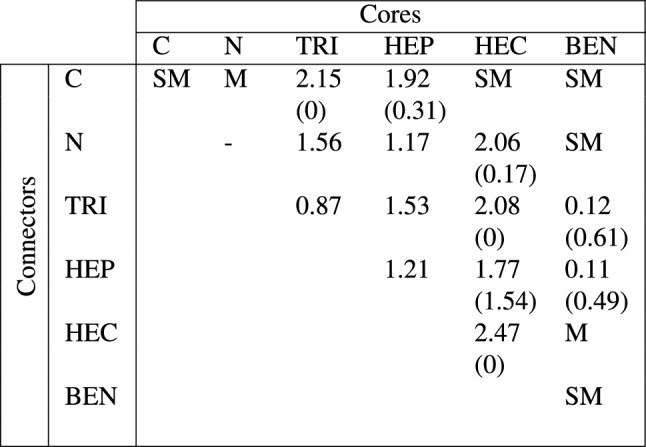
The energy gap of each GCN is reported in eV unit where each column and row are core and connector components, respectively. The spin-up (x) and spin-down (y) energy gaps are reported in a x (y) form if they are discrepant but are reported in a single value if they are the same. The electronic behaviors are denoted as M for metals and SM for semi-metals.

#### C-

Particularly, C atoms in graphene spend their three valence electrons to form $\sigma$ bonds and one valence electron to form conjugated $\pi$ bonds with the entire structure. Lieb^10^ has shown that the net spin is not vanished if numbers of atoms in two sublattices of a bipartite lattice such as graphene are not equal. (Ovchinnikov^53^ has proposed the same equation of the net spin for hydrocarbons). The theorem was validated by an existence of magnetic moment in defective and hydrogenated graphenes^13,14^. In this case, C–C is a perfect lattice graphene where the C atoms in two sublattices are equal, so it is non-magnetic.

For C–N (equivalent to N–C in Table 2) which also has a honeycomb structure, lone pairs of N atoms and lone electrons of C atoms form $\pi$ conjugation without defects suppressing the intrinsic magnetism even the $n_e$ per the unit cell is odd. This is according to the PDOS of CN (see Fig. S3 in Supplementary Information) that the $p_z$ orbitals of C and N atoms dominating the states across the $\hbox {E}_F$. It is because the $p_z$ electrons delocalize and pair together across several cells even the $n_e$ in the unit cell is odd.

#### TRI- and HEP-

**Figure 2:**
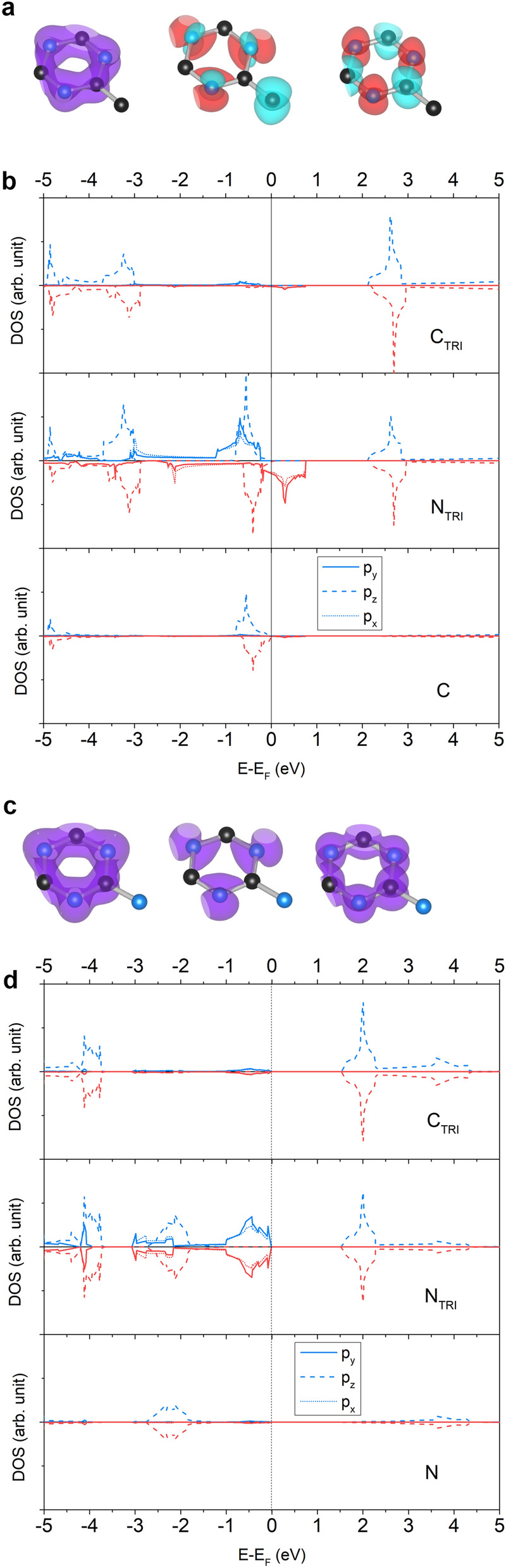
Structures of (**a**) TRI-C and (**c**) TRI-N are illustrated where left, middle, and right figures show isosurfaces of charge density at deep, valence and conduction levels, respectively. The spin-up, spin-down and total chage densities are shown in blue, red, and purple, respectively. The spin-up (blue) and spin-down (red) PDOS of (**b**) TRI-C and (**d**) TRI-N are projected on $p_x$ (dottedline), $p_y$ (solid line) and $p_z$ (solid line) orbitals of C and N atoms.

TRI and HEP components have even $n_e$, so they themselves obtain no magnetic moment, and their combinations, TRI–TRI, HEP–TRI, and HEP–HEP, consequently, yield no magnetism (see Table 2). TRI-N and HEP-N, whose each N connector leaves one lone pair, are also non-magnetic. In contrast to TRI-C and HEP-C, the C connector has one $n_e$ which is the $p_z$ electron granting a magnetic moment to the whole structures. If that is the case, why does the $p_z$ electron of the C connector not delocalize and pair with $p_z$ electrons of TRI? Because the $p_z$ states of the C connector and $\hbox {C}_{\text {TRI}}$ atom are in different energy levels and both are in the same energy levels with $p_z$ states of $\hbox {N}_{\text {TRI}}$ atom (see Fig. 2). This is also true in HEP-C, TRI-N and HEP-N. For TRI-N and HEP-N, the $p_z$ electrons of the N connector already form a lone pair, so the levels mismatch does not matter.

For the electronic property, TRI–TRI and TRI-N are semiconductors with energy gaps 0.87 and 1.56 eV, respectively, while TRI-C is a half-metal with spin-up energy gap 2.15 eV (see Table 3). For TRI-C, the $p_x$ and $p_y$ states of $\hbox {N}_{\text {TRI}}$ atoms dominate the states around the $\hbox {E}_F$, and the $p_z$ states of $\hbox {N}_{\text {TRI}}$ and the C connector are at a bit below the $\hbox {E}_F$. The $p_z$ states of $\hbox {C}_{\text {TRI}}$ and $\hbox {N}_{\text {TRI}}$ atoms hybridize with one another at below − 3 eV which is at different energy level with the $p_z$ states of the C connector (see Fig. 2b). Noting that they appear at the same energy level below − 5 eV, but that is deep from the $\hbox {E}_F$. This result also holds for TRI-N where the $p_z$ states of the N connector hybridize with that of $\hbox {N}_{\text {TRI}}$ atoms at − 2 eV but not with that of $\hbox {C}_{\text {TRI}}$ atoms (see Fig. 2d). For TRI–TRI, since its core and connector are the same, main features of its PDOS are similar to TRI of TRI-C and TRI-N that the $p_x$ and $p_y$ states dominate at valence states near the $\hbox {E}_F$ while the $p_z$ states appear at below − 4 eV (see Fig. S4 in Supplementary Information). Besides, in TRI-C and TRI-N, the $p_z$ states of $\hbox {N}_{\text {TRI}}$ appear two times; one hybridizes with the $p_z$ states of the connector, and another one hybridizes with the $p_z$ states of $\hbox {C}_{\text {TRI}}$ atoms.

Mataga^54^ has proposed that the electrons of atoms in the ring (TRI) are paired through the $\pi$ conjugated bonds leaving their non-bonding $\sigma$ orbitals and non-bonding $\pi$ orbital of the C connector to be unpaired. This is in accordance to our result that the spin moment comes from the valence states of the $p_x$ and $p_y$ states of atoms in the ring and the $p_z$ states of the C connector. The $p_z$ states form a narrow band as in Mataga’s discussion, but the $p_x$ and $p_y$ states form a wide band.

**Figure 3:**
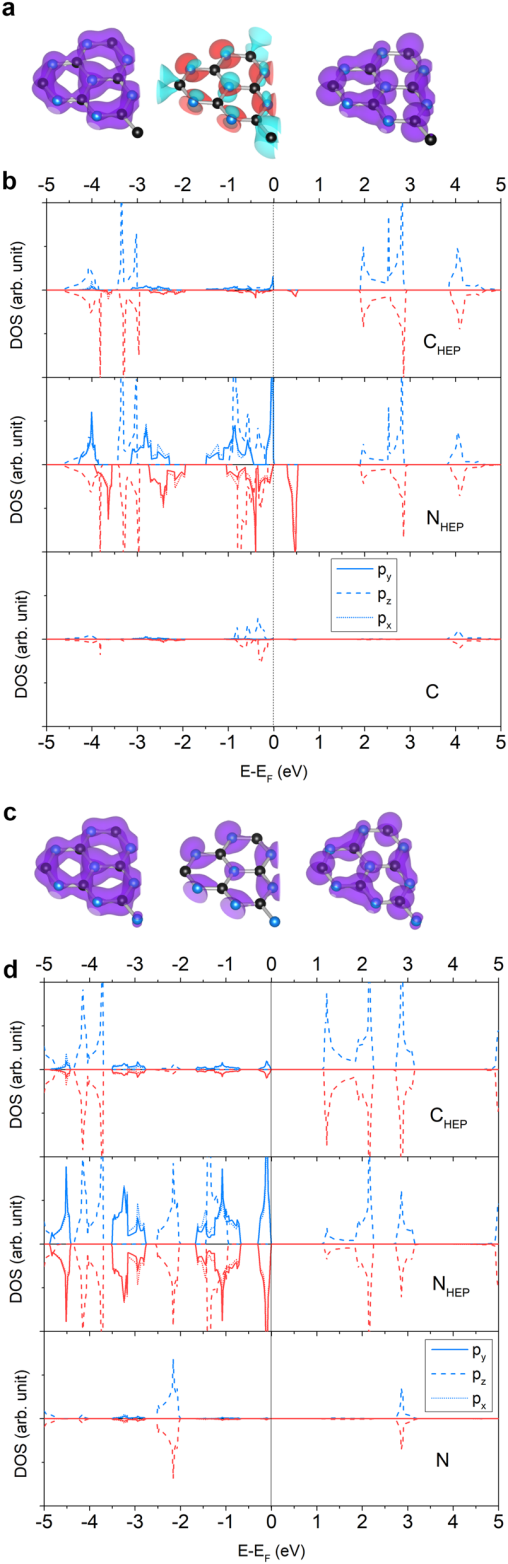
Structures of (**a**) HEP-C and (**c**) HEP-N are illustrated where left, middle, and right figures show isosurfaces of charge density at deep, valence and conduction levels, respectively. The spin-up, spin-down and total chage densities are shown in blue, red, and purple, respectively. The spin-up (blue) and spin-down (red) PDOS of (**b**) HEP-C and (**d**) HEP-N are projected on $p_x$ (dottedline), $p_y$ (solid line) and $p_z$ (solid line) orbitals of C and N atoms.

The HEP-X (X = N, TRI, and HEP) structures whose each component obtains even $n_e$ are non-magnetic semiconductors with 1.17, 1.53, and 1.21 eV for energy gaps, respectively (see Table 3). Since HEP-C is magnetic, it has two energy gaps which are 1.92 and 0.31 for spin-up and spin-down, respectively. The PDOS of HEP-C (HEP-N) shown in Fig. 3 demonstrates the hybridization between atoms in the core and connector similar to that of TRI-C (TRI-N). For HEP-N, the $p_x$ and $p_y$ DOS between spin-up and spin-down are the same for HEP-N. In contrast, for HEP-C, the feature peak of spin-down DOS shift to above the $\hbox {E}_F$ causing the discrepancy between its spin-up and spin-down DOS. This feature also happens if the PDOS of TRI-C and TRI-N are compared. For HEP–TRI and HEP–HEP (see Figs. S5 and S6 in Supplementary Information), the $p_z$ states of atoms in the core and connector appear at the same energy level below − 2 eV while their $p_x$ and $p_y$ states appear near the $\hbox {E}_F$.

#### HEC-

**Figure 4:**
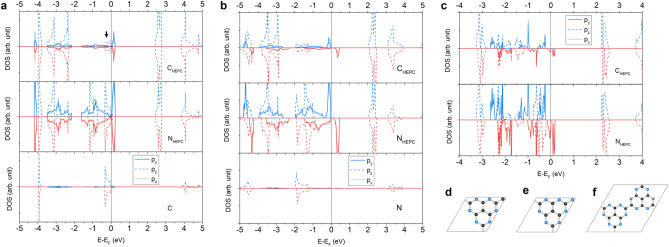
The spin-up (blue) and spin-down (red) PDOS of (**a**) HEC-C, (**b**) HEC-N, and (**c**) HEC–HEC are projected on $p_x$ (dottedline), $p_y$ (solid line) and $p_z$ (solid line) orbitals of C and N atoms. A black arrow points the $p_z$ DOS of $\hbox {C}_{\text {HEC}}$. Structures of (**d**) HEC-C, (**e**) HEC-N, and (**f**) HEC–HEC are illustrated.

The electronic properties of HEC-X (X = C, N, TRI, HEP, and HEC) shown in Table 3 are various where HEC-C is a semimetal. HEC–TRI and HEC–HEC are a half-metal with 2.08 and 2.47 eV, respectively, for spin-up energy gap. HEC-N (HEC–HEP) is a semiconductor where its spin-up and spin-down energy gaps are 2.06 (1.77) and 0.17 (1.54) eV, respectively. Figure 4d–f show the structures of HEC-C, HEC-N, and HEC–HEC, respectively.

The HEC component has odd $n_e$; therefore, its combination with the C connector, HEC-C, is non-magnetic while its combinations with N, TRI, and HEP components are magnetic with one $\mu _B$ per unit cell. On the one hand, the PDOS of HEC-N (see Fig. 4b) shares similar features to that of TRI-C and HEP-C where the $p_x$ and $p_y$ spin-up (spin-down) states are at valence maximum (conduction minimum) level and the $p_z$ states are at deeper energy level. On the other hand, the PDOS of HEC-C shows a distinguish feature that the $p_z$ states of every atom appear at the valance maximum level while their $p_x$ and $p_y$ states shift to conduction minimum level. Because HEC-C has a number of electrons smaller than HEP-N by two electrons, so the $\hbox {E}_F$ which is a function of number of electrons shifts down from above to below the $p_x$ and $p_y$ PDOS peaks (see Fig. 4a). Moreover, $\hbox {C}_{\text {HEC}}$ atoms contribute their $p_z$ states to the valence maximum level in contrast to previous phases, so the $p_z$ orbitals of $\hbox {C}_{\text {HEC}}$ and $\hbox {N}_{\text {HEC}}$ atoms and the C connector can form conjugated $\pi$ bonds delocalizing the electrons and suppressing the intrinsic magnetism. For HEC–TRI and HEC–HEP (see Figs. S7 and S8 in Supplementary Information), their PDOS share similar features to HEP–TRI and HEP–HEP, but they are magnetic.

Despite that HEC–HEC has even $n_e$, it has a magnetization for 2$\mu _B$ per unit cell. Here, we run into the second question that why does the reason for the absence of intrinsic magnetism discussed above not be able to use with HEC–HEC? As aforementioned, for TRI, HEP, and HEC components, the $p_z$ states of their C atoms are at deep energy level while the $p_z$ states of their N atoms appear twice, first at shallow level and second at the energy level as C atoms. Even though the $\pi$ conjugation in HEC–HEC occurs at energy below − 1 eV, only $p_z$ states of $\hbox {N}_{\text {HEC}}$ atoms have a contribution near the $\hbox {E}_F$ (see Fig. 4c). The electrons in the valence maximum level hence do not form conjugated $\pi$ bonds, but localize at the $p_x$ and $p_y$ states of $\hbox {N}_{\text {HEC}}$ atoms. HEC–HEC thus obtains 2$\mu _B$ per unit cell, one of which comes from each HEC component, and the most discrepancy between spin-up and spin-down DOS is from the $p_x$ and $p_y$ states.

#### BEN-

The BEN component has odd $n_e$, so BEN-C and BEN–BEN are non-magnetic, and BEN-X (X = N, TRI, and HEP, HEC) are magnetic with one $\mu _B$ per unit cell except for BEN–HEC which has the magnetization about 0.45$\mu _B$ per unit cell (see Table 2). For the electronic property, BEN–TRI (BEN–HEP) is a semiconductor with spin-up and spin-down energy gap about 0.12 (0.11) and 0.61 (0.49) eV, respectively, BEN–HEC is a metal, and others are semimetals (see Table 3).

**Figure 5:**
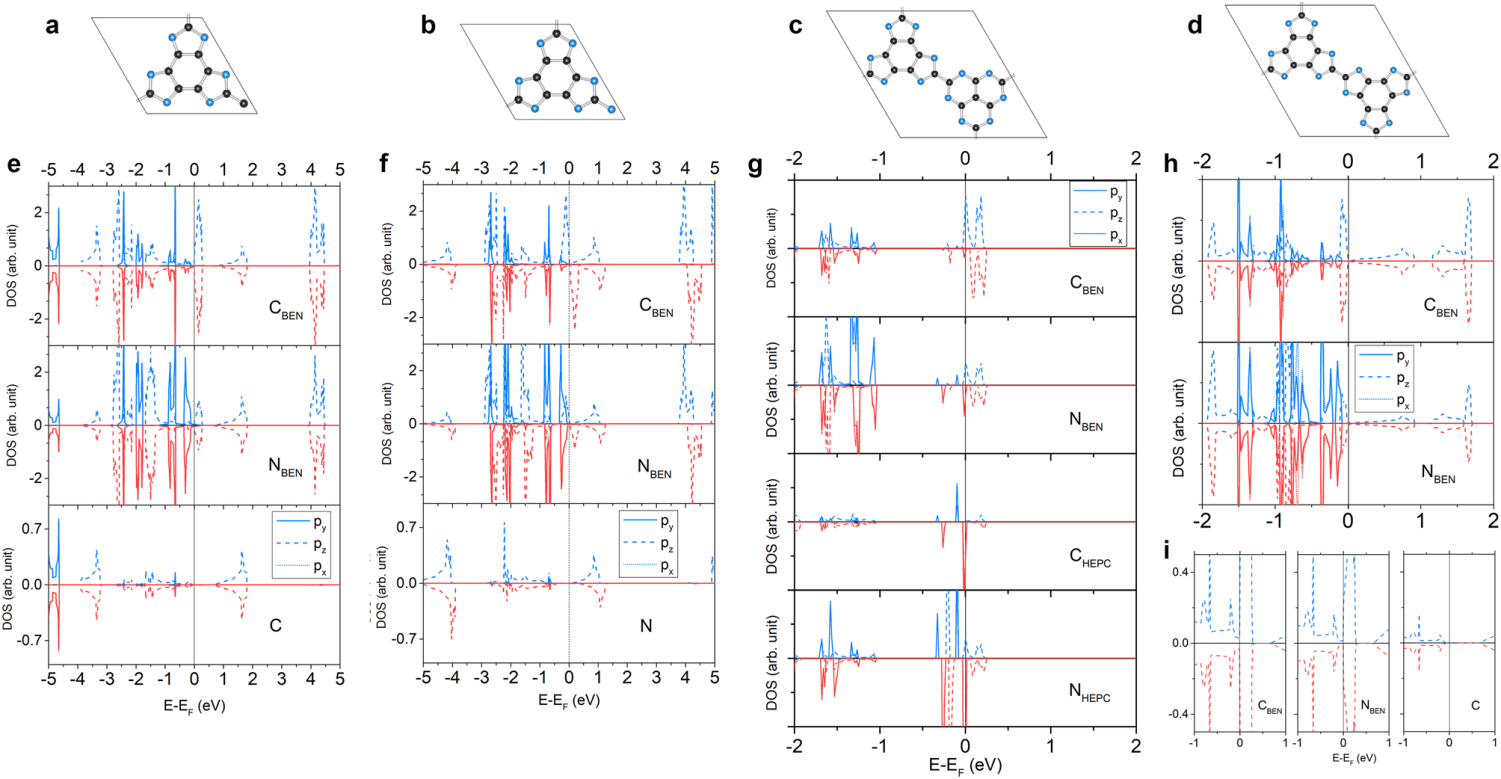
Structures of BEN-X (X = (**a**) C, (**b**) N, (**c**) HEC, and (**d**) BEN) are illustrated. The spin-up (blue) and spin-down (red) PDOS of BEN-X (X = (**e**) C, (**f**) N, (**g**) HEC, and (**h**) BEN) are projected on $p_x$ (dotted line), $p_y$ (solid line) and $p_z$ (solid line) orbitals of C and N atoms. (**i**) The $p_z$ DOS of BEN-C is magnified.

The BEN component contains a C hexagonal ring and three pyrroles (see Fig. 5a–d for structures of BEN-X for X = C, N, HEC, and BEN, respectively). Its PDOS shows that the $p_x$ and $p_y$ states are at energy level lower than the $p_z$ states (see Fig. 5h). Near the $\hbox {E}_F$, the $p_z$ DOS of $\hbox {C}_{\text {BEN}}$ atoms and the $p_x$ and $p_y$ DOS of $\hbox {N}_{\text {BEN}}$ atoms are high while DOS of connector C_{TRI,HEP,HEC}_ atoms are tiny. Consequently, the valence states of BEN–BEN are dominated by the $p_z$ states of $\hbox {C}_{\text {BEN}}$ atoms, and the $p_x$ and $p_y$ states of $\hbox {N}_{\text {BEN}}$ atoms appear at lower energy level (see Fig. 5h). For BEN-C, it is non-magnetic, so there is no discrepancy between spin-up and spin-down DOS (see Fig. 5e). Because its number of electrons is smaller than BEN–BEN, so its $\hbox {E}_F$ drops below the $p_z$ states causing the $p_x$ and $p_y$ states are at the valence maximum level. Although, the $p_z$ states of $\hbox {C}_{\text {BEN}}$ and $\hbox {N}_{\text {BEN}}$ atoms and the C connector appear from the $\hbox {E}_F$ down to − 4 eV showing a sign of the $\pi$ conjugation but its PDOS near the $\hbox {E}_F$ is tiny (see Fig. 5i).

For BEN-N, the $p_z$ states show the discrepancy between spin-up and spin-down DOS while the $p_x$ and $p_y$ states are slightly discrepant between spin-up and spin-down DOS (see Fig. 5f). Therefore, a spin moment of BEN-N comes from electrons in the $p_z$ states in contrast to, e.g., TRI-C and HEP-C that their spin moments come from electrons in the $p_x$ and $p_y$ states of N atoms. Because BEN-N has a number of electrons higher than BEN-C by one electron, so the $\hbox {E}_F$ shifts to higher than the $p_z$ states becoming the valence maximum. For BEN–TRI and BEN–HEP, their PDOS features near the $\hbox {E}_F$ are from the $p_z$ states which is clearly induced by the BEN component (see Figs. S9 and S10 in Supplementary Information). The $p_z$ states of $\hbox {C}_{\text {TRI}}$ atoms show no contribution here while those of $\hbox {C}_{\text {HEP}}$ atoms have small contributions. The $p_x$ and $p_y$ states of $\hbox {N}_{\text {TRI}}$ and $\hbox {N}_{\text {HEP}}$ atoms are discrepant in contrast to the PDOS of phases discussed in previous sections. The spin moments of BEN–TRI and BEN–HEP are also from the electrons in the $p_z$ states which is similar to BEN-N.

For BEN–HEC, its PDOS is a mixing between the characteristics of BEN and HEC components (see Fig. 5g). At the $\hbox {E}_F$, the spin-up and spin-down DOS are from the $p_z$ states and the $p_x$ and $p_y$ states, respectively, where the former is dominated by the PDOS of $\hbox {C}_{\text {BEN}}$ atoms, and the latter is dominated by the PDOS of $\hbox {N}_{\text {HEC}}$ and $\hbox {C}_{\text {HEC}}$ atoms. The $p_z$ states at the $\hbox {E}_F$ are actually from the PDOS of every atom except $\hbox {C}_{\text {HEC}}$ atoms, so the $\pi$ conjugation does not occur at this level but at below -1.0 eV. Therefore, BEN and HEC components obtain different spin moments where the spin moment of the BEN component is greater.

#### Summary

The building blocks can be grouped by their PDOS characteristics into three groups which are the atoms, i.e., C and N atoms, the carbon nitride rings, i.e., TRI, HEP, and HEC components, and BEN component. The individual atoms contribute their $p_z$ states as their valence maximum states. The carbon nitride rings have their valence maximum states dominated by the $p_x$ and $p_y$ states of N atoms, and their C and N atoms form $\pi$ bonds at deeper energy level. The BEN component has the $p_z$ states of C atoms as its valence maximum states and the $p_x$ and $p_y$ states of N atoms at the energy level next to the valence maximum states. If the $\pi$ conjugation forms across the core and the connector of GCNs near the $\hbox {E}_F$, the intrinsic magnetism of GCNs will be suppressed. Otherwise, for a structure with odd $n_e$, the magnetic moment will be localized in the $p_x$ and $p_y$ states ($p_z$ states) of N (C) atoms if the structure is a combination with the carbon nitride rings (BEN component).

Besides, there are three structures which are C–N, HEC–HEC, and BEN–HEC that their magnetism cannot be determined by counting the $n_e$. The C–N is non-magnetic because of the $\pi$ conjugation at the valence maximum level. The HPEC–HEC and BEN–HEC, in contrast, lack of the $\pi$ conjugation at the valence maximum level, so their magnetic moments are localized at each component to the system.

Two factors thereupon playing the important role to raise or demote the intrinsic magnetism of GCNs are the $n_e$ and the $\pi$ conjugation. The components with odd (even) $n_e$ tend to contribute the unpaired (paired) electrons to the system. Their combinations mostly result in presence and absence of the intrinsic magnetism if the $n_e$ in the unit cell is odd and even, respectively. For the odd-odd combinations, i.e., C–C, HEC-C, and BEN-C, the $p_z$ orbitals of the core and connector form the conjugated $\pi$ bonds pairing electrons across the structures. For the odd-even combinations, e.g., TRI-C, HEP-C, and HEC-N, the $\pi$ conjugation does not occur because the $p_z$ states of the C and N connectors are at the energy level same as (different from) the $p_z$ states of N (C) atoms of the cores. The $p_z$ states of N and C atoms of the cores are also hybridizing at the same energy level but at deeper level. In the even-even case, it does not matter if there are conjugated $\pi$ bonds near the $\hbox {E}_F$. The electrons in each component are paired and contribute no magnetic moment.

### Alloys

**Figure 6:**
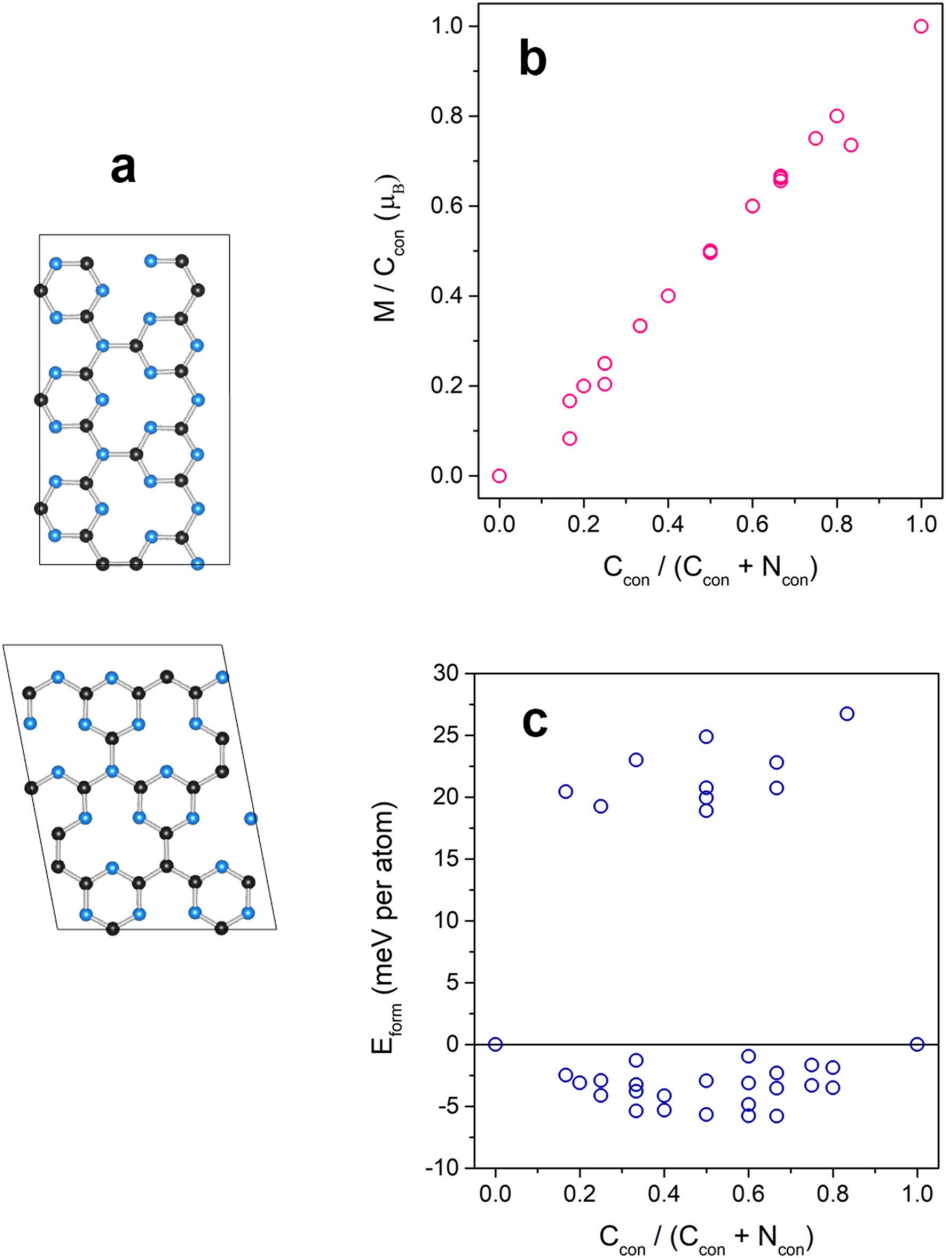
(**a**) Some order phases of TRI-C/N alloy are illustrated for a demonstration. (**b**) Magnetizations and (**c**) the formation energy of order phases compared with the energy of TRI-N and TRI-C are plotted with respect to the ratio between a number of $\hbox {C}_{{con}}$ and a number of all connectors where $\hbox {C}_{{con}}$ and $\hbox {N}_{{con}}$ are C and N connectors. Noting that 0.0 and 1.0 in x-axis represent TRI-N and TRI-C, respectively.

The g-$\hbox {C}_3\hbox {N}_4$ or TRI-N has been synthesized while the g-$\hbox {C}_4\hbox {N}_3$ or TRI-C has yet existed only in the simulation^27^. TRI-C is magnetic, but its $E_{form}$ is 133 meV higher than TRI-N (see Table 1). To achieve the intrinsic magnetism, the connectors are alloyed between C and N atoms, so the magnetization can be arisen proportional to a number of C connectors ($\hbox {C}_{{con}}$). The structures of the alloy can be represented by different ordered patterns of C and N connectors created up to 42 atoms per primitive cell for 52 structures (including TRI-C and TRI-N) by using Hart and Forcade’s algorithm^55^ where some of order structures are illustrated in Fig. 6a.

Figure 6b is a plot of ratio between the magnetization and the number of $\hbox {C}_{{con}}$ in an ordered structure with respect to a ratio between $\hbox {C}_{{con}}$ and total number of the connectors in the ordered structure. As expected, the magnetization of order structure increases linearly as the number of $\hbox {C}_{{con}}$ in the structure increasing because each $\hbox {C}_{{con}}$ contributes a radical to the system. Figure 6c shows that the formation energy of the ordered solid solutions is below zero with respect to those of TRI-N and TRI-C indicating that the connector C and N atoms are likely to be chemically ordered at low temperature in thermodynamic equilibrium. The transition temperature between order-disordered phase that helps to verify a structure found in the experiment, for instance, HEP/TRI-C/N^56^, hence needs to be investigated^19^, but it is beyond the scope of this work.

### Hydrogenation

The intrinsic magnetism of GCNs can be altered by a hydrogenation^39^. Therefore, an atom in each GCN is here passivated by one H atom per unit cell in order to investigate the change in magnetism and the thermodynamically stability. Each GCN has several atomic positions for H atom to passivate, for example, H atom can passivate on $\hbox {C}_{\text {TRI}}$, $\hbox {N}_{\text {TRI}}$, and the C connector of TRI-C which are 3 configurations in total. As a result, the configurations of each GCNs except C–N, that the H atom is passivating with N atoms, have the $E_{form}$ lower than their non-hydrogenated phases. The H atom passivating with C atom of GCNs yields high $E_{form}$ because it induces high strain on the structure while the passivation with N atom is on the side of the structure inducing less strain (see Fig. S11 in Supplementary Information).

Since different configurations yield different results in the magnetizations and the $E_{form}$, Table 4 shows the magnetization of the configuration of each hydrogenated GCN that has the lowest $E_{form}$. Noting that the magnetization of hydrogenated graphene (C–C) is one $\mu _B$ per unit cell, but it is not reported in Table 4 because its $E_{form}$ is higher than graphene. For hydrogenated HEC-X, the HEC component whose $\hbox {N}_{\text {HEC}}$ atom is passivated by H atom has even $n_e$, so hydrogenated HEC-X obtains one $\mu _B$ of magnetization per unit cell for X = C and HEC and zero magnetization for X = N, TRI, and HEP.

For hydrogenated BEN-X, the HEC component whose $\hbox {N}_{\text {HEC}}$ atom is passivated by H atom has also even $n_e$, so hydrogenated BEN-X obtains zero magnetization for X = N, TRI, and HEP. Accordingly, the magnetization of hydrogenated BEN–HEC is one $\mu _B$ per unit cell because one of its components has even $n_e$ while another has odd $n_e$, so the magnetic moments between BEN and HEC are no more annihilated in the hydrogenated case. In contrast, the magnetization of hydrogenated BEN-C is 0.20$\mu _B$ per unit cell even its $n_e$ is odd because its valence electrons can be shared through the $\pi$ conjugation at the valence maximum states.

For hydrogenated TRI-X, the TRI component whose $\hbox {N}_{\text {HEC}}$ atom is passivated by H atom has odd $n_e$, so hydrogenated TRI-X obtains zero magnetization for X = C and 0.84 and 1 $\mu _B$ per unit cell for X = N and TRI, respectively. Notwithstanding that hydrogenated HEP-X for X = N, TRI, and HEP obtain odd $n_e$, their thermodynamically favorable configurations yield zero magnetization.

**Table 4:**
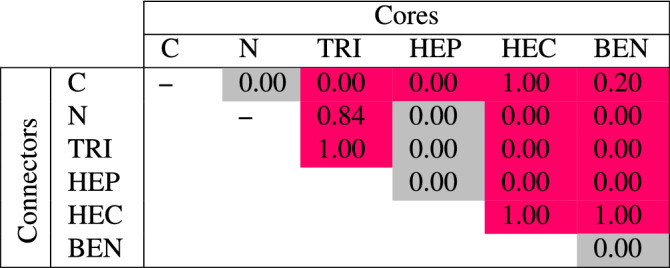
The magnetization of each hydrogenated GCN is reported in $\mu _B$ unit where each column and row are core and connector components, respectively. The pink (grey) labels the phase that its magnetization does (not) different from its non-hydrogenated phase.

## Conclusions

Firstly, we present building blocks comprising 19 GCNs (and graphene) and the reason behind their emergent intrinsic magnetism through bonding states and charge density. The structure that has even $n_e$ is non-magnetic because all valence electrons are paired suppressing the magnetic moment. The structure that has odd $n_e$ can be either magnetic or non-magnetic depending on the valence maximum states are localized or delocalized, respectively. The valence maximum states are ensured to be localized if they are from the $p_x$ and $p_y$ orbitals but are not necessary to be delocalized if they are from the $p_z$ orbitals. The $p_z$ orbitals of every atom in the core and connector must present in the valence maximum states to form the $\pi$ conjugation; otherwise, the unpaired electron will be localized in its component (core or connector) producing the magnetic moment.

Secondly, we also apply the building-blocks scheme to study alloys mixing between g-$\hbox {C}_3\hbox {N}_4$ and g-$\hbox {C}_4\hbox {N}_3$. The magnetization of the alloy increases linearly as a concentration of the C connector, and some promising alloys are energetically favorable. Lastly, a H atom attached on GCN lowers the $E_{form}$ of the structure. The H atom can change the magnetization of the system depending on a position it is passivating. Therefore, these understandings could lead to the future design for stable GCNs that maintain to be radicals with magnetism.

## Methods

The VASP package^57,58^ used to perform a first principle calculation based on spin-polarized density functional theory (DFT) has been employed to study the structural, electronic, and magnetic properties of GCNs. In the spin-polarized DFT, the charge density and the magnetization density can be written as^59^

1 $$\begin{aligned} n(\mathbf{r }) = \sum _\alpha n^{\alpha \alpha (\mathbf{r })},\;\;\; \mathbf{m }(\mathbf{r }) = \sum _{\alpha \beta } \pmb {\sigma }^{\alpha \beta }n^{\alpha \beta }(\mathbf{r }), \end{aligned}$$

where $\pmb {\sigma }$ is $(\sigma _x, \sigma _y, \sigma _z)$, a vector of the Pauli matrices and $\alpha$ and $\beta$ are spin indices, spin up ($\uparrow$) or spin down ($\downarrow$). The collinear spin-polarization treats the spins to be aligned in the same direction, typically *z* direction, so $\pmb {\sigma } = (0, 0, \sigma _z)$. The spin density matrix is thus

2 $$\begin{aligned} n^{\alpha \beta }({\mathbf {r}}) = \frac{1}{2}(n({\mathbf {r}})\delta ^{\alpha \beta } + m_z({\mathbf {r}})\sigma _z^{\alpha \beta }). \end{aligned}$$

Therefore, the charge density and magnetization density, respectively, can be computed by

3 $$\begin{aligned} n({\mathbf {r}}) = n^{\uparrow }({\mathbf {r}}) + n^{\downarrow }({\mathbf {r}}), \end{aligned}$$

4 $$\begin{aligned} m({\mathbf {r}}) = n^{\uparrow }({\mathbf {r}}) - n^{\downarrow }({\mathbf {r}}). \end{aligned}$$

where $n^{\uparrow }$ and $n^{\downarrow }$ are eigenvalues of the spin density matrix solved by the Kohn–Sham equation^60^.

The DFT calculation is used the projector augmented wave (PAW) method^61^ for a pseudopotential and the Perdew–Berke–Erzenhof (PBE) for an exchange-correlation functional^62^. All calculations have been performed by including the van der Waals correction using Grimme DFT-D3 method^63^. The energy cutoff is 600 eV, and the k-point interval has been set to be 0.02 Å$^{-1}$ at most for every size of unit cell. The *c*-axis has been constrained to be 20 Å in order to avoid an interaction between layers. The tetrahedron method^64^ has been performed to calculate the spin-polarized density of states (DOS).

The formation energy ($E_{form}$) of carbon nitrides and hydrogenated carbon nitrides systems per atom has been calculated using

5 $$\begin{aligned} E_{form} (\text {C}_x\text {N}_y\text {H}_z) = \frac{E(\text {C}_x\text {N}_y\text {H}_z) - 0.5[xE(\text {C}_2) + yE(\text {N}_2) + zE(\text {H}_2)]}{x+y+z}, \end{aligned}$$

where $E(\text {C}_x\text {N}_y\text {H}_z)$ is the energy of $\text {C}_x\text {N}_y\text {H}_z$, and $E(\text {C}_2)$, $E(\text {N}_2)$, and $E(\text {H}_2)$ are the energy of graphene, $\hbox {N}_2$ molecule, and $\hbox {H}_2$ molecule, respectively.

## Supplementary Information

Supplementary Information.
