# Supplementary material for: Presence and absence of intrinsic magnetism in graphitic carbon nitrides designed through C–N–H building blocks
Source: Sci Rep. 2022 Feb 11;12:2343. doi: 10.1038/s41598-022-05590-4 (PMC8837644; doi:10.1038/s41598-022-05590-4)
Supplement: Supplementary file 1 — Supplementary Information. [file 41598_2022_5590_MOESM1_ESM.pdf]

Supplementary Information: Presence and absence of intrinsic magnetism in graphitic carbon nitrides designed through C-N-H building blocks

Teerachote Pakornchote[1,2], Annop Ektarawong[1,2], Akkarach Sukserm[1,2], Udomsilp Pinsook[1,2], and Thiti Bovornratanaraks[1,2,*]

[1]Extreme Conditions Physics Research Laboratory, Physics of Energy Materials Research Unit, Department of Physics, Faculty of Science, Chulalongkorn University, Bangkok, Thailand
[2]Thailand Center of Excellence in Physics, Commission on Higher Education, 328 Si Ayutthaya Road, Bangkok, 10400, Thailand
[*]thiti.b@chula.ac.th

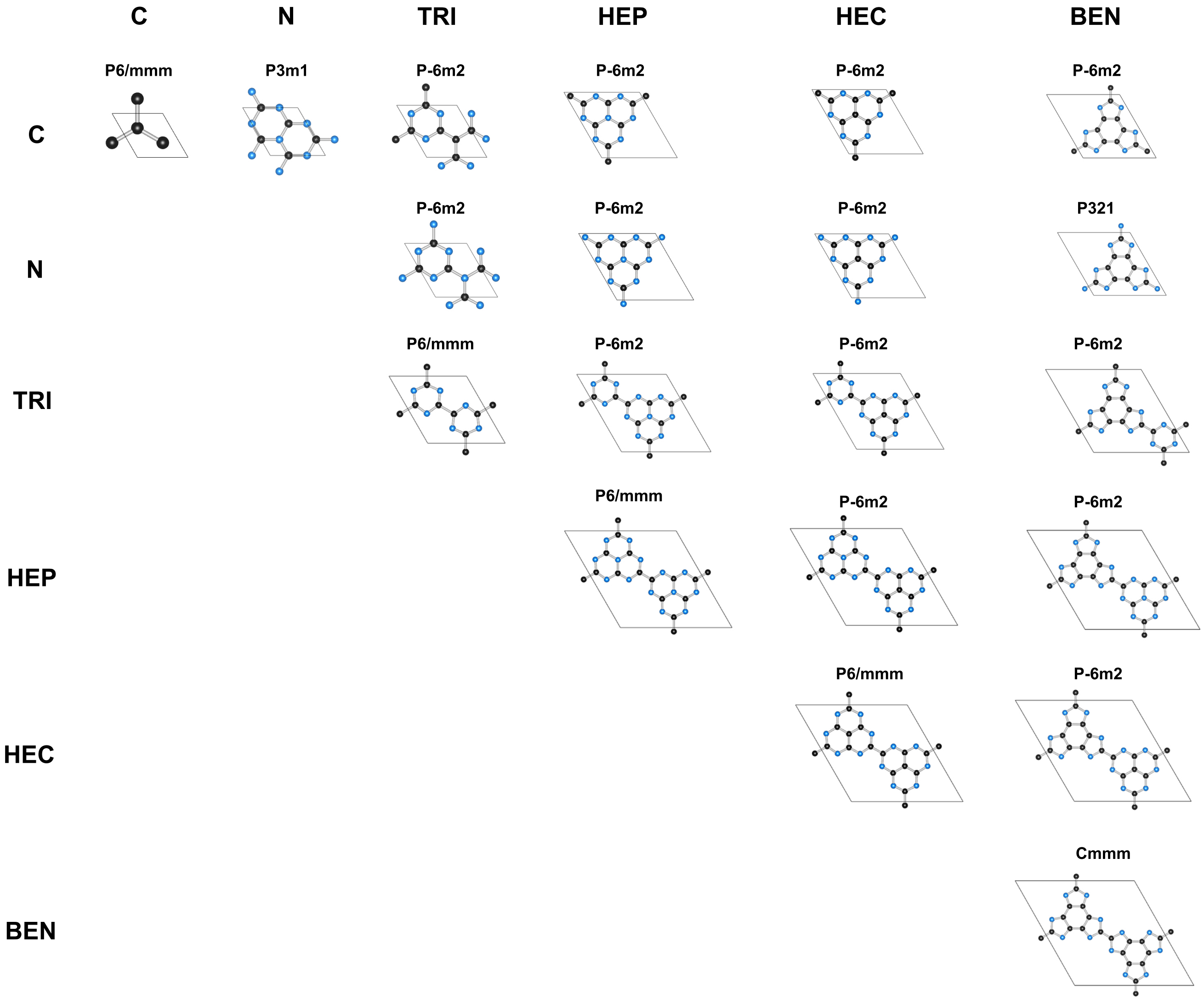

Figure S1. 19 GCN structures and graphene after relaxation and their space groups are presented. C-N and BEN-N have three-fold symmetry, BEN-BEN has two-fold symmetry, and others have six-fold symmetry.

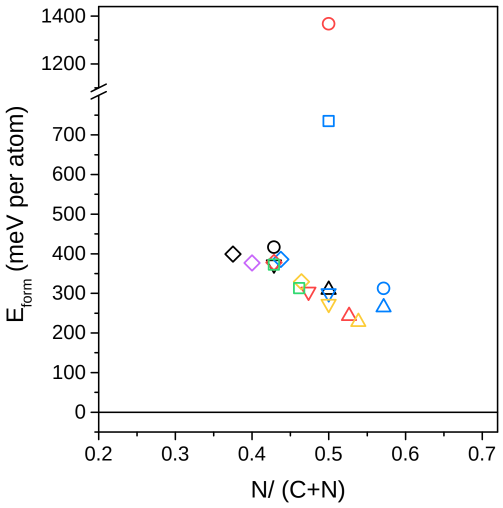

Figure S2. The formation energies of GCNs with respect to the magnetization are presented. The symbols and their colors represent core and connector of the phases, respectively. The cores are C (square), TRI (circle), HEP (triangle), HEC (down triangle) and BEN (diamond). The connectors are C (black), N (blue), TRI (red), HEP (yellow), HEC (green) and BEN (purple). The star symbol denotes N2 molecule.

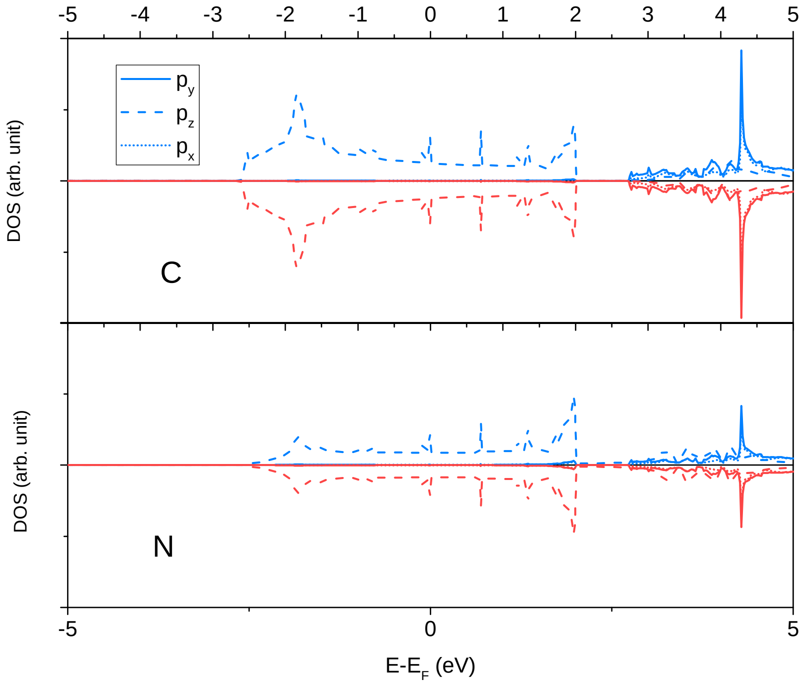

Figure S3. PDOS of C-N.

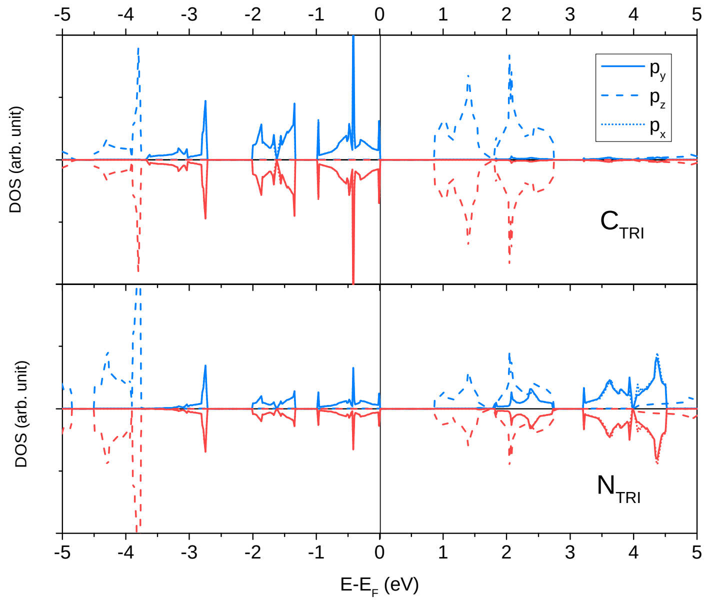

Figure S4. PDOS of TRI-TRI.

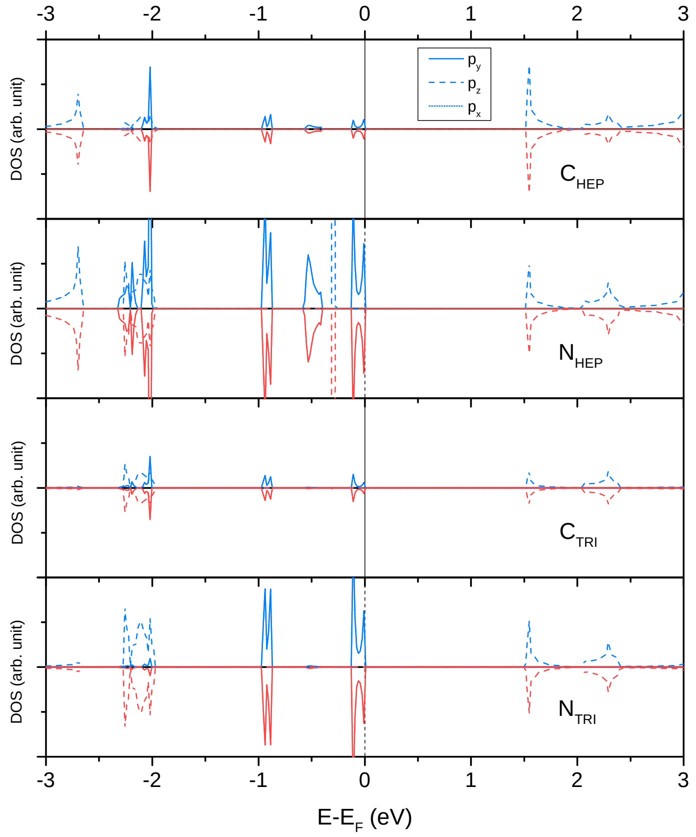

Figure S5. PDOS of HEP-TRI.

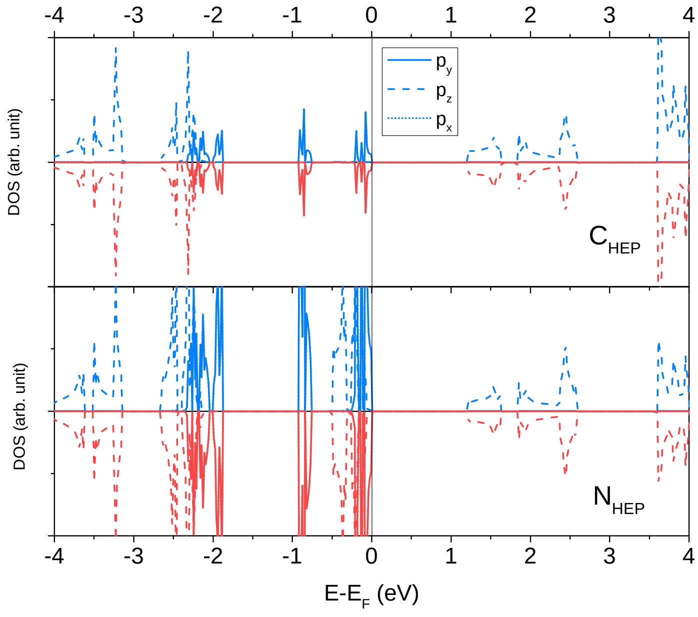

Figure S6. PDOS of HEP-HEP.

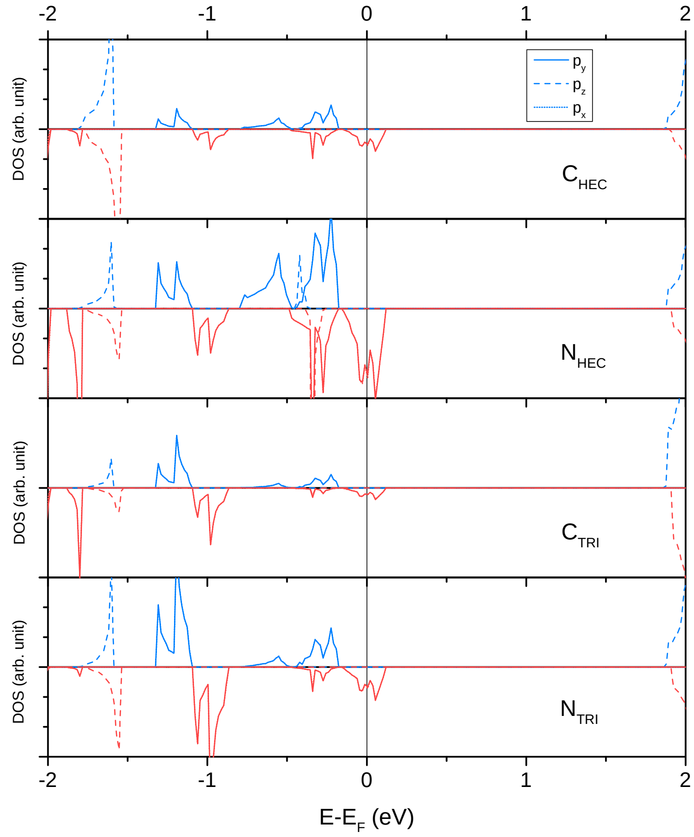

Figure S7. PDOS of HEC-TRI.

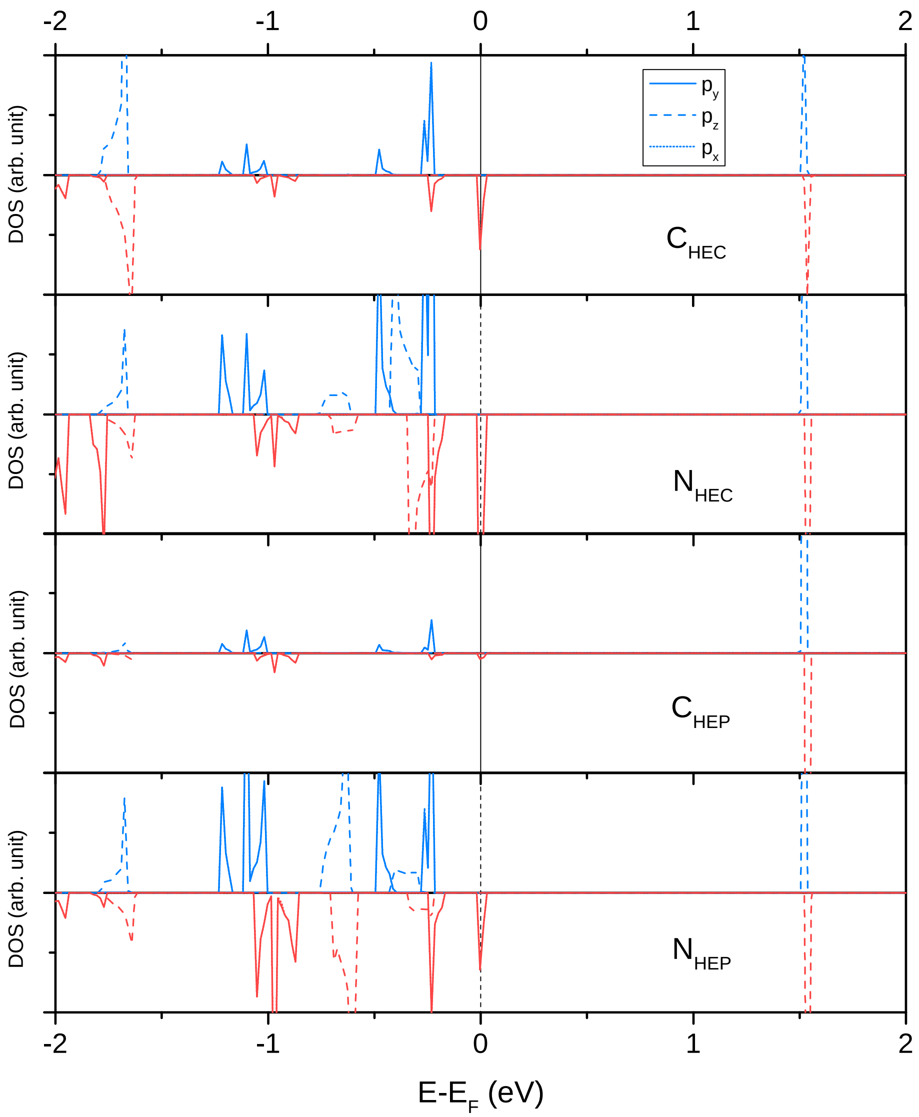

Figure S8. PDOS of HEC-HEP.

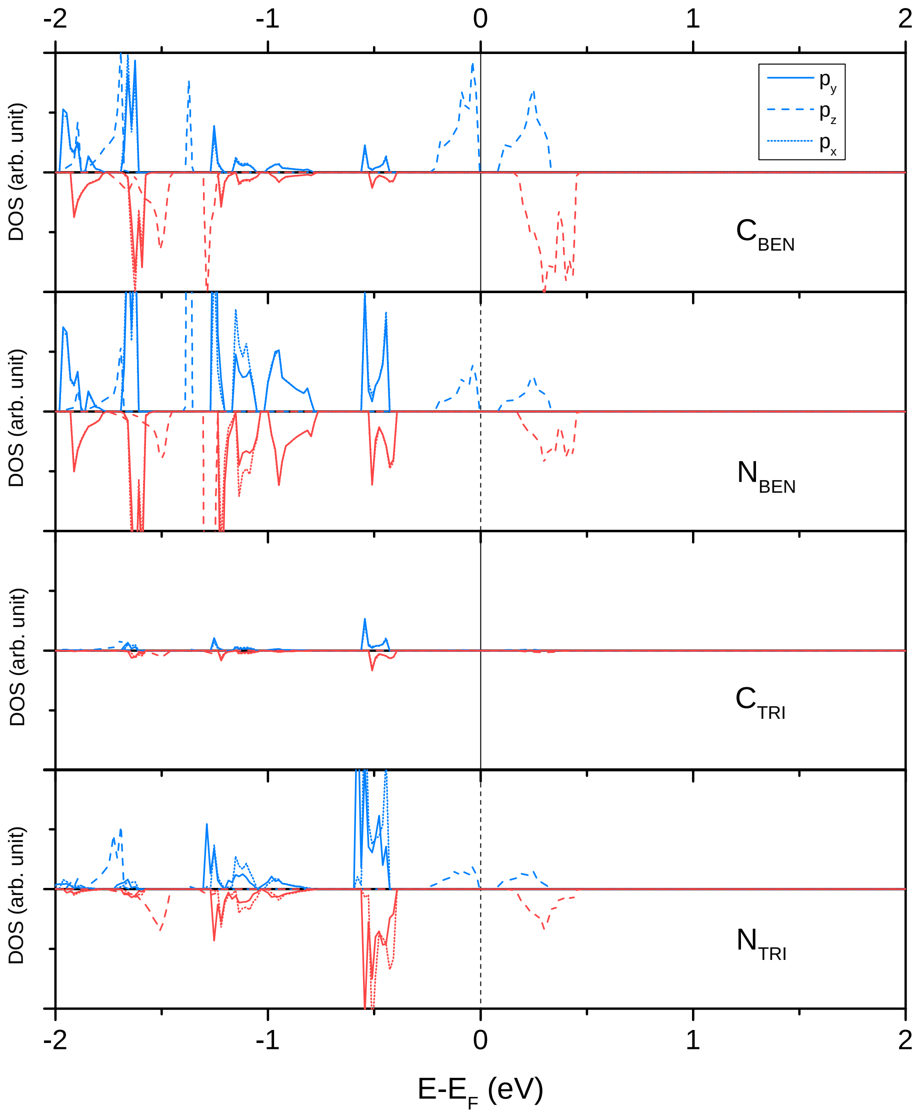

Figure S9. PDOS of BEN-TRI.

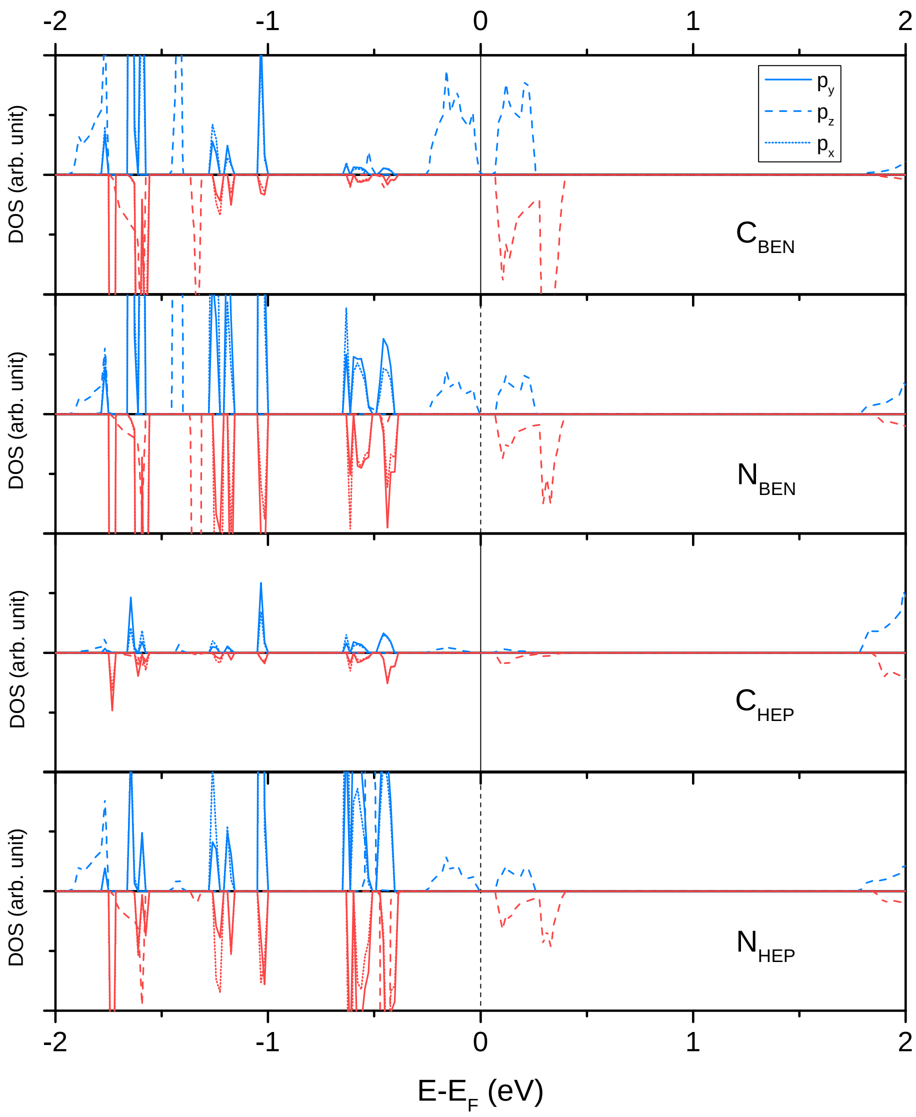

Figure S10. PDOS of BEN-HEP.

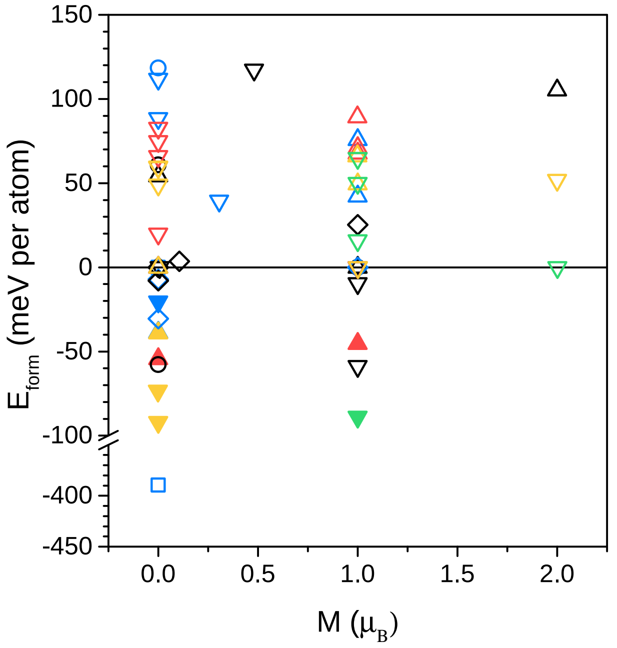

Figure S11. The formation energies of hydrogenated GCNs with respect to the magnetization are presented. The symbols and their colors represent core and connector of the phases, respectively. The cores are C (square), TRI (circle), HEP (triangle), HEC (down triangle) and BEN (diamond). The connectors are C (black), N (blue), TRI (red), HEP (yellow), HEC (green) and BEN (purple). The open and solid symbols are the phases whose H atom is attached to C and N atoms, respectively.
